# CNNDLP: A Method Based on Convolutional Autoencoder and Convolutional Neural Network with Adjacent Edge Attention for Predicting lncRNA–Disease Associations

**DOI:** 10.3390/ijms20174260

**Published:** 2019-08-30

**Authors:** Ping Xuan, Nan Sheng, Tiangang Zhang, Yong Liu, Yahong Guo

**Affiliations:** 1 School of Computer Science and Technology, Heilongjiang University, Harbin 150080, China; 2 School of Mathematical Science, Heilongjiang University, Harbin 150080, China; 3 School of Information Science and Technology, Heilongjiang University, Harbin 150080, China

**Keywords:** lncRNA-disease association prediction, feature learning based on convolutional autoencoder, convolutional neural networks, attention at adjacent edge level, similarity calculation based on multiple bipartite networks

## Abstract

It is well known that the unusual expression of long non-coding RNAs (lncRNAs) is closely related to the physiological and pathological processes of diseases. Therefore, inferring the potential lncRNA–disease associations are helpful for understanding the molecular pathogenesis of diseases. Most previous methods have concentrated on the construction of shallow learning models in order to predict lncRNA-disease associations, while they have failed to deeply integrate heterogeneous multi-source data and to learn the low-dimensional feature representations from these data. We propose a method based on the convolutional neural network with the attention mechanism and convolutional autoencoder for predicting candidate disease-related lncRNAs, and refer to it as CNNDLP. CNNDLP integrates multiple kinds of data from heterogeneous sources, including the associations, interactions, and similarities related to the lncRNAs, diseases, and miRNAs. Two different embedding layers are established by combining the diverse biological premises about the cases that the lncRNAs are likely to associate with the diseases. We construct a novel prediction model based on the convolutional neural network with attention mechanism and convolutional autoencoder to learn the attention and the low-dimensional network representations of the lncRNA–disease pairs from the embedding layers. The different adjacent edges among the lncRNA, miRNA, and disease nodes have different contributions for association prediction. Hence, an attention mechanism at the adjacent edge level is established, and the left side of the model learns the attention representation of a pair of lncRNA and disease. A new type of lncRNA similarity and a new type of disease similarity are calculated by incorporating the topological structures of multiple bipartite networks. The low-dimensional network representation of the lncRNA-disease pairs is further learned by the autoencoder based convolutional neutral network on the right side of the model. The cross-validation experimental results confirm that CNNDLP has superior prediction performance compared to the state-of-the-art methods. Case studies on stomach cancer, breast cancer, and prostate cancer further show the ability of CNNDLP for discovering the potential disease lncRNAs.

## 1. Introduction 

For the past few years, genetic information has been thought to be stored only in protein-coding genes, while non-coding RNAs (ncRNAs) are only byproducts of the transcription process [1,2]. However, accumulating evidences indicate that ncRNAs play important roles in various biological processes, especially long non-coding RNAs (lncRNAs), with lengths > 200 nucleotides [3,4]. 

The previous methods have been presented for predicting the lncRNA-disease associations, and they are classified into three categories. The methods in the first category utilize machine learning methods to identify the candidate associations. Chen et al. develop a semi-supervised learning model, LRLSLDA, which uses Laplacian regularized least squares to identify possible associations between lncRNA and disease [5]. A model based on the Bayesian classifier was developed for predicting candidate disease lncRNAs [6]. However, most of the methods in this category fail to achieve the good performances for the lncRNAs with no any known associated diseases. 

The second category of methods takes use of the biological premise that lncRNAs with similar functions tend to be associated with similar diseases [7]. First, the similarity between two lncRNAs is calculated by the diseases associated with the lncRNAs, and a network composed of lncRNA is constructed by using the similarities between lncRNAs [8]. Several methods are presented for predicting the lncRNAs related to a given disease based on the lncRNA network, for instance, via random walks on the lncRNA network [9,10] or by utilizing the information of neighboring nodes of lncRNA [11]. These methods are ineffective for the new diseases with no known related lncRNAs, as they rely on a set of seed lncRNAs that have been observed to be related to the disease. Some methods attempt to introduce additional information about diseases to solve this shortcoming. Disease information is incorporated with the lncRNA network to create a heterogeneous lncRNA-disease network that contains information of lncRNA similarities, that of disease similarities and that of lncRNA-disease associations. Several methods exploit the information, but they construct different models within the heterogeneous network to estimate the association scores between the lncRNAs and the diseases. For instance, the association scores are derived by random walks in the lncRNA-disease network [10,12], or by matrix factorization of lncRNA-disease associations [13,14]. Since lncRNAs are often involved in disease processes along with miRNAs, it is necessary to integrate the interactions and associations about the miRNAs. Nevertheless, most of the previous methods overlook these information related to the miRNAs.

The third category of methods integrates multiple biological data sources about the lncRNA, the miRNA, the proteins. MFLDA integrates various information about the genes and the miRNAs interacted with lncRNAs, and about the diseases associated with lncRNAs. The method constructs a matrix factorization based on data fusion model for predicting disease lncRNAs [15]. Zhang et al. introduce the protein information to establish the lncRNA-protein-disease network and predict the candidate associations between lncRNAs and diseases based on propagating information streams in the network [16]. The diverse information available about the lncRNAs, diseases, genes, and proteins reflect the associations of lncRNAs and diseases from the different perspectives. However, it is difficult for these methods to deeply integrate heterogeneous data from multiple sources. Therefore, we present a novel prediction method based on dual convolutional neural networks to learn the latent representations of lncRNA-disease pairs from the multiple-source data.

## 2. Experimental Evaluations and Discussions

### 2.1. Evaluation Metrics

Five-fold cross-validation is used to evaluate the prediction performances of CNNDLP and several state-of-the-art methods for predicting lncRNA-disease associations. All the known lncRNA–disease associations are regarded as positive samples, and the unobserved associations are taken as negatives samples. We randomly divided all the positive samples into five subsets, and four of them are used to training the model. As the number of positive samples is far less than that of the negative samples (ratio of positive samples to negative samples is nearly 1:36 in our study), during the training process, we select the negative samples randomly whose number match to the number of the positive training samples, and these negative samples are also used for training the model. The positive samples in the remaining subset and all the negative samples are considered as the testing samples. The number of positive samples and that of negative samples during the cross-validation process are listed in Supplement Table S1. In particular, during each cross-validation, the positive samples used for testing are removed and the lncRNA similarities are recalculated by using the remaining positive samples. 

We obtain the association scores of testing samples and prioritize them by their scores. The positive samples are ranked higher, which indicate that the prediction performance is better. The lncRNA-disease node pairs whose scores are greater than a classification threshold θ are identified as positive samples, and the ones that have lower scores are determined as negative samples. The true positive rates (TPRs) and the false positive rates (FPRs) at various θ values are calculated as follows:

(1) $$TPR=\frac{TP}{TP+FN},\;FPR=\frac{FP}{FP+TN}$$

where TP and TN are the numbers of positive and negative samples that are identified correctly, while FN and FP are the numbers of misidentified positive and negative samples. The receiver operating characteristic (ROC) curve can be drawn according to the TPRs and FPRs at each various θ, while the area under the ROC curve (AUC) is usually used to evaluate the overall performance of a prediction method [17].

A serious imbalance between the positive samples and the negative ones appears since their ratio is 1:36. For such imbalanced cases, precision-recall (PR) curve is confirmed to be more informative than ROC curve [18]. Therefore, the PR curve is used as another important measurement for the prediction performance of each method. Precision and recall are calculated as follows:

(2) $$precision=\frac{TP}{TP+FP},\;recall=\frac{TP}{TP+FN}$$

where *precision* is the proportion of the real positive samples among the samples that are identified as the positive ones, while *recall* is the proportion of the real positive samples to the total actual positive ones. The area under the P-R curve (AUPR) is also utilized to evaluate the performance of lncRNA-disease association prediction [19]. 

In addition, the top candidate lncRNAs are usually selected by the biologists for further experimental verification of their associations with an interested disease. Therefore, we demonstrate the recall rates of the top 30, 60, and 240 candidates, which demonstrates how many of the positive samples are identified correctly within the ranking list of top *k*.

### 2.2. Comparison with Other Methods

To assess the prediction performance of CNNDLP, we compare it with several state-of-the-art methods for predicting disease lncRNAs: SIMCLDA [20], Ping’s method [21], MFLDA [15], LDAP [22] and CNNLDA [8]. CNNDLP and the other four methods have specific hyperparameters for fine-tuning to achieve their best association prediction performance. We choose the values of CNNDLP’s hyperparameters, α, *β* and λ, from {0.1, ..., 0.9}. CNNDLP achieved the best performance of five-fold cross-validation, when *α* = 0.9, *β* = 0.8 and *λ* = 0.3. The prediction performances of CNNDLP at different values of *α, β*, and *λ* on CNNDLP in the Supplementary Table S2, Supplementary Table S3, and Supplementary Table S4. In addition, the window size of all convolutional layers and pooling layers in CNNDLP is set as 2 × 2. The number of filters in the first and the second convolutional layers $n_{conv1}$ and $n_{conv2}$ are set to 16 and 32 respectively. CNNDLP has a great many parameters, which is easy to make the model overfit all the training samples. Therefore, we adopt dropout strategy and batch normalization to prevent the overfitting. To make a fair comparison, we set the hyperparameters of other methods to the optimal values that are recommended by their respective literatures (i.e., *α_l_* = 0.8, *α_d_* = 0.6 and *λ* = 1 for SIMCLDA, *α* = 0.6 for Ping’s method, *α* = 105 for MFLDA, *Gap open* = 10. *Gap extend* = 0.5 for LDAP).

**Figure 1:**
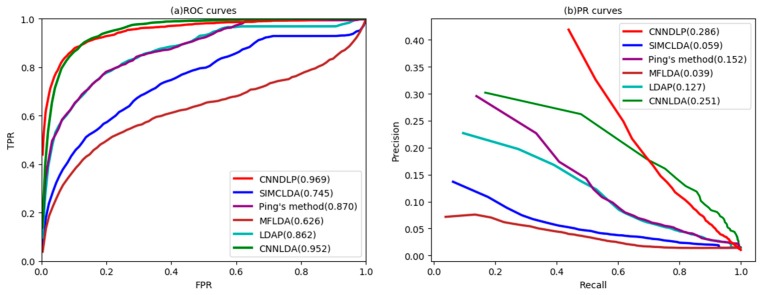
ROC curves and PR curves of CNNDLP and other methods for all diseases.

As shown in Figure 1a, CNNDLP yields the highest average performance on all of the 405 diseases (AUC = 0.969). In particular, its performance is increased SIMCLDA by 21.2%, Ping’s method by 9.3%, MFDDA by 34.4%, LDAP by 10.7%, and CNNLDA by 1.7%. The AUCs of the five methods on 10 well-characterized diseases are also listed in Table 1, and CNNDLP achieves the best performance in all of the 10 diseases. The AUC of CNNDLP is slightly better than CNNLDA, but the AUPR of the former is 3.5% higher than the latter. The possible reason for this is that CNNLDA did not learn the low-dimensional network representation of a lncRNA-disease pair. Ping’s method and the LDAP achieved decent performance as they take advantage of the various similarities of different types of lncRNAs and diseases. MFLDA does not perform as well as the other four methods. A possible reason is that it ignored the lncRNA similarity and the disease similarity, while are exploited by the other methods. The improvement of CNNDLP over the compared methods is primarily due to the fact that it deeply learns the attention representation and low-dimensional network-level representation of the lncRNA-disease node pairs. 

As shown in Figure 1b, CNNDLP’s average AUPR is also higher than other methods on 405 diseases (AUPR = 0.286). Its average AUPR is 22.7%, 13.4%, 24.7%, 15.9%, and 3.5% higher SIMCLDA, Ping’s method, MFLDA, LDAP and CNNLDA, respectively. In addition, CNNDLP performs the best performance among nine of the ten well-characterized diseases (Table 2).

**Figure 2:**
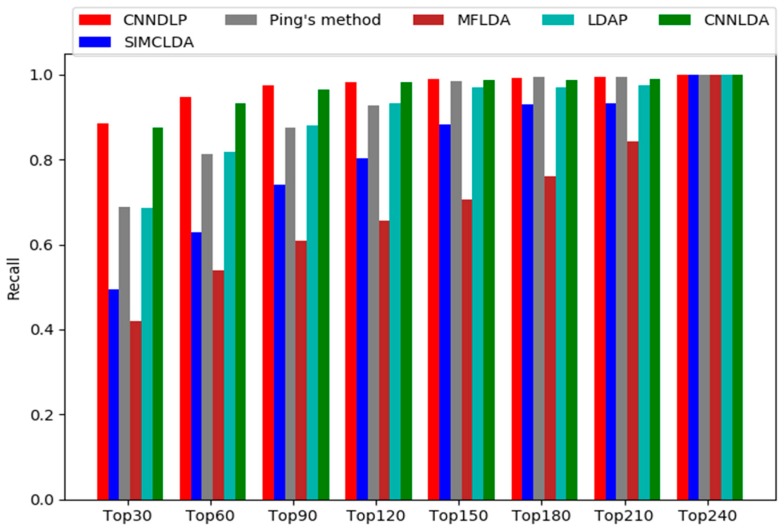
Recall values of top *k* candidates of CNNDLP and other four methods.

A higher recall value in the top *k* of ranking list indicates that more real lncRNA-disease associations are identified correctly. Figure 2 shows CNNDLP outperforms the other methods at different top *k* cutoffs, and ranks 88.6% in top 30, ranks 94.6% in top 60, ranks 97.5% in top 90, and ranks 98.3% in top 120. Most of the recall rates of Ping’s method are very close to LDAP. The former ranked 68.9%, 81.3%, 87.5% and 92.7% in top 30, 60, 90 and 120, respectively, and the latter ranked 68.5%, 81.7%, 88.0% and 93.3%. MFLDA is still worse than the other methods, and it ranked 42.0%, 53.9%, 61.0% and 65.6%.

In addition, a paired Wilcoxon test is conducted to confirm whether CNNDLP’s prediction performance is significantly greater than the other methods. The statistical results in Table 3 show that CNNDLP yields better performance than the other methods in terms of not only AUCs but AUPRs, as well for the threshold *p-*value of 0.05.

### 2.3. Case Studies: Stomach Cancer, Breast Cancer and Prostate Cancer

To further demonstrate the capability of CNNDLP to discover potential disease-related candidate lncRNAs, we construct the case studies on stomach cancer, breast cancer, and prostate cancer. For each of these three diseases, we prioritize the candidate lncRNA-disease associations based on their association scores and gather their respective 15 candidates.

Stomach cancer is currently the fourth most common malignant tumor in the world and the second leading cause of cancer-related death [23]. First, Lnc2Cancer is a manually curated database that are verified associations between the lncRNAs and the human cancers by the biological experiments [24]. Twelve of 15 candidates are included by Lnc2Cancer (Table 4), which indicates that these lncRNAs are indeed associated with the disease.

Second, LncRNADisease records more than 4564 lncRNA-disease associations that are obtained from experiments, the published literatures or computation, and then the dysregulation of lncRNAs are manually confirmed [25]. There are 14 candidates contained by the LncRNADisease, indicating they are upregulated or downregulated in stomach cancer tissues. In addition, one candidate labeled by “literature” is supported by the literature, and it is confirmed to have dysregulation in the cancer when compared with the normal tissues [26].

Among the top 15 candidates for breast cancer, 11 candidates are reported in Lnc2Cancer with abnormal expression in breast cancer. (Table 5) LncRNADisease contains 12 candidates, which confirms the associations between these candidates and the disease. The remaining 2 candidates are confirmed by the literatures to have desregulation in the breast cancer [27,28].

The top 15 prostate cancer-related candidates and the corresponding evidences are listed in Table 6. Fourteen candidates are included by Lnc2Cancer and 14 ones are contained by LncRNADisease, which indicates that they truly are related to the disease. All the case studies confirm that CNNDLP is effective and impactful for discovering potential candidate disease lncRNAs.

### 2.4. Prediction of Novel Disease lncRNAs

After five-fold cross validation and case studies to confirm its prediction performance, we further apply CNNDLP to 405 diseases. All the known lncRNA-disease associations are used for training CNNDLP’s to predict potential disease-related lncRNAs. The top 50 potential candidates for each of 405 diseases are demonstrated in Supplementary Table S5.

## 3. Materials and Methods

### 3.1. Datasets for lncRNA-Disease Association Prediction

We obtained thousands of lncRNA-disease associations, lncRNA-miRNA interactions and miRNA-disease associations from a published work [15]. The human lncRNA-disease database (LncRNADisease) consists of 2687 lncRNA-disease associations that were verified by the biological experiments, covering 240 lncRNAs and 405 diseases [29]. The disease similarities were calculated based on directed acyclic graphs (DAGs) and the DAGs were constructed based on the disease terms from the U.S. National Library of Medicine (MeSH). The 1002 lncRNA-miRNA interactions were originally extracted from starBasev2.0 and they have been confirmed by biological experiments [30], and were involved 495 miRNAs. The 13,559 miRNA-disease associations were obtained from HMDD database [31].

### 3.2. Bipartite Graphs about the lncRNAs, Diseases, miRNAs, and Representations

**Figure 3:**
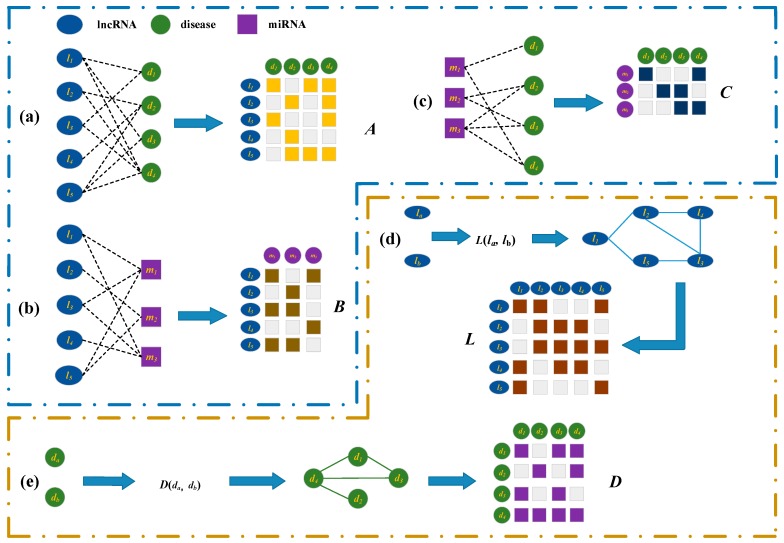
Construction and representation of multiple bipartite graphs. (**a**) Construct a lncRNA-disease association bipartite graph based on the known associations between lncRNAs and diseases, and its’ matrix representation ***A***. (**b**) Construct lncRNA-miRNA interactions bipartite graph based on the known lncRNA-miRNA interactions, and its’ matrix representation ***B***. (**c**) Construct miRNA-disease association bipartite graph based on known miRNA-disease associations, and its’ matrix representation ***C***. (**d**) Calculate the lncRNA similarity, and construct the matrix representation ***L***. (**e**) Calculate the disease similarity, and construct the matrix representation ***D***.

We firstly construct a bipartite graph composed of lncRNAs and diseases by connecting them according to the observed lncRNA-disease associations (Figure 3a). The graph is represented by matrix $A=\left[A_{ij}\right]\in R^{N_{l}\times N_{d}}$, where $N_{l}$ and $N_{d}$ are the number of lncRNAs and that of diseases, respectively. Each of rows corresponds to a lncRNA while each of columns represent a disease. If a lncRNA $l_{i}$ has been observed to be associated a disease $d_{j}$, the $A_{ij}$ in A is set to 1, otherwise $A_{ij}$ is 0.

There are a great many interactions between the lncRNAs and miRNAs that have been confirmed by the biological experiments [32]. A bipartite graph composed of lncRNA and miRNA nodes is established when there are known interactions between them (Figure 3b). $B=\left[B_{ij}\right]\in R^{N_{l}\times N_{m}}$ is used to represent interaction matrix, the graph including $N_{l}$ lncRNAs and $N_{m}$ miRNAs. If it is known that lncRNA $l_{i}$ is interacted with miRNA $m_{j}$, $B_{ij}=1$, or $B_{ij}=0$ when their interaction has not been observed.

An edge is added to connect a miRNA and a disease, when they are observed to have association (Figure 3c). $C=\left[C_{ij}\right]\in R^{N_{m}\times N_{d}}$ is a matrix representing a bipartite graph with $N_{m}$ miRNAs and $N_{d}$ diseases. We set $C_{ij}$ to 1 if miRNA $m_{i}$ is associated with disease $d_{j}$, or 0 when no such association is observed.

### 3.3. LncRNA-Disease Association Prediction Model Based on CNN

In this section, we describe the prediction model based on convolutional neural networks and attention mechanism for learning the latent representation and predicting the association score of lncRNA $l_{i}$ and disease $d_{j}$. The embedding layer is firstly constructed by incorporating the associations, the similarities, the interactions about lncRNAs, diseases, miRNAs. A novel prediction model is constructed and it is composed of the left and right parts. The left side of the model learns the attention representation of $l_{i}$ and $d_{j}$, while the network representation of $l_{i}$ and $d_{j}$ is learned in the right side of model. Each of the two representations goes through a fully connected layer and a softmax layer and the associated possibility between $l_{i}$ and $d_{j}$ is obtained and it is regarded as their association score. The final score is the weighted sum of two association scores.

#### 3.3.1. Embedding Layer on the Left

##### lncRNA Functional Similarity Measurement

On the basis of the biological premise that lncRNAs with similar functions are more possibly to be associated with similar diseases, the similarity of two lncRNAs is measured by their associated diseases. For instance, lncRNA $l_{a}$ is associated with disease $d_{1}$, $d_{2}$ and $d_{4}$ and lncRNA $l_{b}$ is associated with diseases $d_{2}$, $d_{4}$ and $d_{5}$. The similarity between $E_{a}=\left\{d_{1},d_{2},d_{4}\right\}$ and $E_{b}=\left\{d_{2},d_{4},d_{5}\right\}$ is regarded as the functional similarity of $l_{a}$ and $l_{b}$. The lncRNA similarity that are used by us is calculated according to the Xuan’s method [8]. Matrix $L=\left[L_{ij}\right]\in R^{N_{l}\times N_{l}}$ is the lncRNA similarity matrix (Figure 3d), where $L_{ij}$ is the similarity of lncRNAs $l_{i}$ and $l_{j}$, $L_{ij}$ value changes between 0 and 1.

##### Disease Similarity Measurement

All semantic terms related to a disease form its directed acyclic graph (DAG). The semantic similarities between the diseases are successfully calculated by Wang et al. based on their DAGs [33]. We calculate the disease similarities according to Wang’s method, and the similarities can be represented by matrix $D=\left[D_{ij}\right]\in R^{N_{d}\times N_{d}}$, where $D_{ij}$ is the similarity of disease $d_{i}$ and $d_{j}$ (Figure 3e). The similarity of two diseases also varies between 0 and 1.

##### The Left Embedding Layer for Integrating the Original Information

**Figure 4:**
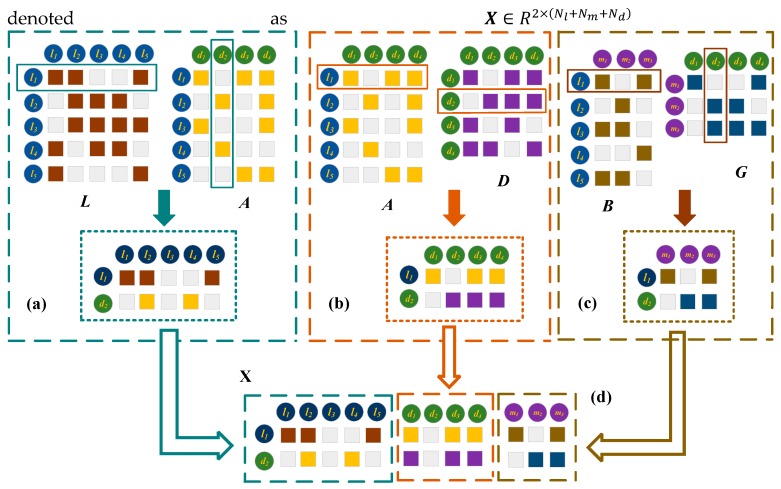
Construction of the left embedding layer matrix of $l_{1}$ and $d_{2}$, X. (**a**) Construct the first part of X by exploiting the lncRNA similarities and the lncRNA-disease associations. (**b**) Construct the second part of X by integrating the lncRNA-disease associations and the disease similarities. (**c**) Construct the third part of X by incorporating the lncRNA-miRNA interactions and the miRNA-disease associations. (**d**) Concatenate the three parts of X.

If a lncRNA and a disease have similarity relationships and association relationships with the more common lncRNAs, they are more likely to associated with each other. We take the lncRNA $l_{1}$ and the disease $d_{2}$ as an example to explain the process of constructing the embedding layer on the left. As shown in Figure 4, let $L_{1}$ represents the first row of L which records the similarities between $l_{1}$ and all the lncRNAs. The second row of $A^{T}$, $A_{2}^{T}$, contains the associations between $d_{2}$ and all the lncRNAs. For example, as $l_{1}$ is similar to $l_{2}$ and $l_{5}$, and $d_{2}$ has been associated with $l_{2}$, $l_{4}$ and $l_{5}$, $l_{1}$ is possibly related to $d_{2}$. We stack $L_{1}$ and $A_{2}^{T}$ together as the first part of the embedding layer. Similarly, $l_{1}$ and $d_{2}$ are more likely to associate when $l_{1}$ and $d_{2}$ have the similarity and association connections with more common diseases. Therefore, we stack $A_{1}$ and $D_{2}$ as the second part of the embedding layer. In addition, when a lncRNA and a disease have interaction and association relationships with the common miRNAs, they are more possibly to have association. For instance, there is a possible association between $l_{1}$ and $d_{2}$, since $l_{1}$ interacts with miRNAs $m_{1}$ and $m_{3}$, and disease $d_{2}$ is associated with $m_{2}$ and $m_{3}$. The first row of B and the second row $G^{T}$ are stacked as the third part of the embedding layer. The final embedding layer matrix between $l_{1}$ and $d_{2}$ is denoted as $X\in R^{2\times \left(N_{l}+N_{m}+N_{d}\right)}$. 

##### Attention at the Adjacent Edge Level

For a lncRNA node or a disease node, not all the adjacent edges of the node have equal contributions for learning the representation of a pair of lncRNA-disease. In order to solve the issue, we establish the attention mechanism to enhance the adjacent edges that are important for predicting the lncRNA-disease associations. In the embedding layer matrix X, $X_{ij}$ represents the connection case between the *i*-th node and the *j*-th node, and $X_{ij}$ is assigned an attention weight $\alpha_{ij}$, which is defined as follows,

(3) $$F_{ij}=tan\;h\left(WX_{ij}+b\right)\;$$

(4) $$\alpha_{ij}=\frac{exp\left(F_{ij}^{T}u_{e}\right)}{\sum_{j}\exp\left(F_{ij}^{T}u_{e}\right)}\;$$

(5) $${\hat{X}}_{ij}=\alpha_{ij}X_{ij\;}$$

where W and b are a weight matrix and a context vector respectively, and $u_{e}$ is a bias vector. $F_{ij}$ is an attention score that represents the importance of $X_{ij}$. $\alpha_{ij}$ is a normalized importance $X_{ij}$. $\hat{X}$ is the enhanced embedding layer matrix after the attention mechanism at the adjacent edge level is applied for X.

#### 3.3.2. Embedding Layer on the Right

**Figure 5:**
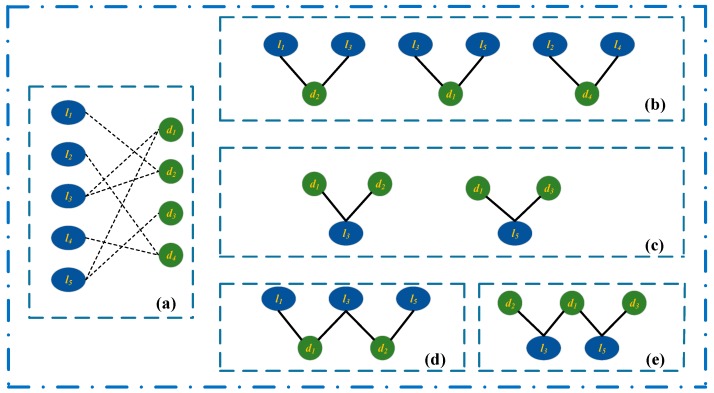
Calculation of the first type of lncRNA similarity and the first type of disease similarity. (**a**) The lncRNA-disease association bipartite network. (**b**) Calculate the lncRNA similarities based on the common associated disease nodes. (**c**) Computer the disease similarities based on their common related lncRNA nodes. (**d**) Calculate the lncRNA similarities according to their associated similar disease nodes. (**e**) The disease similarity calculation based on their related similar lncRNA nodes.

First, it is known that two lncRNA nodes are similar if they are associated with some common disease nodes [22]. In the bipartite network of lncRNA-disease (Figure 5a), lncRNA $l_{1}$ and $l_{3}$ are associated with a common disease node $d_{2}$, so $l_{1}$ and $l_{3}$ are similar. $l_{3}$ and $l_{5}$ are also similar because they are related to a disease node $d_{1}$ (Figure 5b). Similarly, $d_{1}$ is similar to $d_{2}$ as they are associated with common lncRNA node $l_{3}$ (Figure 5c). Second, if two lncRNA nodes have no common neighboring nodes, while they are related to some similar disease nodes, they are also similar to each other [22]. As shown in Figure 5d, $l_{1}$ and $l_{5}$ are similar, because their neighboring nodes $d_{1}$ and $d_{2}$ are similar. Similarly, $d_{2}$ and $d_{3}$ are similar as they are associated with similar neighboring nodes $l_{3}$ and $l_{5}$ (Figure 5e). Ping et al. successfully measured the lncRNA similarities and the disease similarities by utilizing the lncRNA-disease bipartite network.

Unlike Ping’s method that focused on a single bipartite network, multiple kinds of lncRNA similarities and disease similarities are calculated by utilizing the bipartite networks from different sources about lncRNA-disease associations, lncRNA-miRNA interactions and miRNA-disease associations. The first kind of lncRNA similarity $L^{\left(1\right)}=\left[L_{ij}^{\left(1\right)}\right]\in R^{N_{l}\times N_{l}}$, and the first kind of disease similarity $D^{\left(1\right)}=\left[D_{ij}^{\left(1\right)}\right]\in R^{N_{d}\times N_{d}}$ are calculated according to Ping’s method. The second kind of lncRNA similarity is measured by exploiting the information of common miRNA nodes and similar ones interacting with two lncRNA nodes in the lncRNA-miRNA bipartite network, and it is denoted as $L^{\left(2\right)}=\left[L_{ij}^{\left(2\right)}\right]\in R^{N_{l}\times N_{l}}$. Finally, the second kind of disease similarity $D^{\left(2\right)}=\left[D_{ij}^{\left(2\right)}\right]\in R^{N_{d}\times N_{d}}$ is calculated based on the miRNA-disease bipartite network. 

In order to incorporate two kinds of lncRNA similarities $L^{\left(1\right)}$ and $L^{\left(2\right)}$, the final lncRNA similarity $L^{\left(c\right)}$ is defined as follows,

(6) $$L^{\left(c\right)}=\alpha L^{\left(1\right)}+\left(1-\alpha \right)L^{\left(2\right)}$$

where *α* is the parameter utilized to control the contributions of $L^{\left(1\right)}$ and $L^{\left(2\right)}$.

Similarly, the final disease similarity $D^{\left(c\right)}$ is the weighted sum of $D^{\left(1\right)}$ and $D^{\left(2\right)}$, as follows,

(7) $$D^{\left(c\right)}=\beta D^{\left(1\right)}+\left(1-\beta \right)D^{\left(2\right)\;}$$

where *β* is a parameter for regulating the weights of $D^{\left(1\right)}$ and $D^{\left(2\right)}$. 

The right embedding layer for integrating the second kinds of lncRNA and disease similarities. The establishment of the right embedding layer matrix $Y\in R^{2\times \left(N_{l}+N_{m}+N_{d}\right)}$ is similar to the left embedding layer matrix X. First, we stack the first row of $L^{\left(c\right)}$, $L_{1}^{\left(c\right)}$, and $A_{2}^{T}$ together as the first part of the embedding layer. Second, $A_{1}$ and $D_{2}^{\left(c\right)}$ are stacked as the second part of the embedding layer. Finally, the first row of B and the second row $G^{T}$ are stacked as the third part of the embedding layer.

### 3.4. Convolutional Module on the Left 

**Figure 6:**
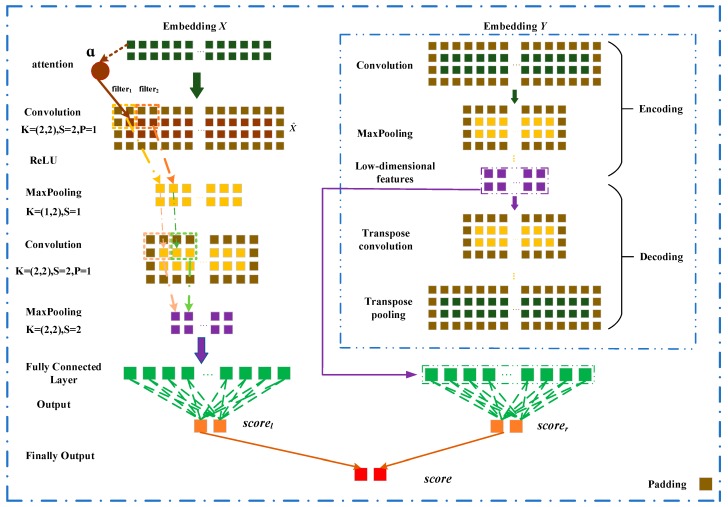
Construction of the prediction model based on the convolutional neural network and convolutional autoencoder for learning the attention representation and the low-dimensional network representation.

In this section, we describe a novel model based on convolutional neural networks with adjacent attention for learning latent representations of lncRNA-disease node pairs. The overall architecture is showed in Figure 6. We describe the left convolutional module in detail. Left module includes convolution and activation layer, max-pooling layer, fully connected layer. The embedding matrix $\hat{X}\in R^{2\times \left(N_{l}+N_{m}+N_{d}\right)}$ is inputted the convolutional module to learn an original representation of a pair of lncRNA-disease node. 

For a convolutional layer, the length and the width of a filter are set to w and h respectively, which means the filter is applied on w×h features. In order to learn the marginal information of the embedding matrix $\hat{X}$, we pad zeros around $\hat{X}$. Let the number of filters is $n_{conv}$. The convolution filters $W_{conv}\in R^{w\times h\times n_{conv}}$ are applied to the embedding matrix $\hat{X}$, and obtain the feature maps $Z\in R^{n_{conv}\times \left(4-w+1\right)\times \left(2+N_{l}+N_{m}+N_{d}-h+1\right)}$. ${\hat{X}}_{ij}$ is the element at the *i*-th row and *j*-th column of $\hat{X}$. ${\hat{X}}_{k,i,j}$ represent a region in a filter when the kth filter slides the position ${\hat{X}}_{ij}$.

(8) $${\hat{X}}_{k,i,j}=\hat{X}\left(i:i+w,j:j+h\right)$$

(9) $$Z_{k}\left(i,j\right)=g\left(W_{k,i,j}*{\hat{X}}_{k,i,j}+b\left(k\right)\right)$$

$$i\in \left[1,\;4-w+1\right]j\in \left[1,\;2+N_{l}+N_{m}+N_{d}-h+1\right],\;k\in \left[1,\;n_{conv}\right]$$

$Z_{k}\left(i,j\right)$ is the element at the *i*-th row and the *j*-th column of the *k-*th feature map. g is relu function that it is a nonlinear activation function [34], $W_{k}$ is the weight matrix of the *k*-th filter and ***b*** is a bias vector. 

The max-pooling layer is used to down-sample the features of the feature maps $Z_{k}\left(i,j\right)$, and it outputs the most important feature in each feature map. Given an input $Z_{k}\left(i,j\right)$, the output of pooling layer is shown as follows,

(10) $$V_{k}\left(i,j\right)=\max\left(Z_{k}\left(i:i+w_{p},j+h_{p}\right)\right)$$

$$i\in \left[1,\;5-w-w_{p}+1\right],j\in \left[1,\;3+N_{l}+N_{m}+N_{d}-h-h_{p}+1\right],k\in \left[1,\;n_{conv}\right]$$

where $w_{p}$ is the length of a filter of pooling layer and $h_{p}$ is the width. $V_{k}\left(i,j\right)$ is the element at the *i*-th row and the *j*-th column in the kth feature map. $\hat{X}$ goes through two convolutional and two max-pooling layers, and we obtain the original representation $Z_{left}$ of $l_{1}$ and $d_{2}$ from the left convolutional module.

Finally, $Z_{left}$ is flattened to a vector z, which z is feed to fully connected layer. A softmax layer is used to normalized the output of the fully connected layer and we have

(11) $$score_{l}=softmax\left(Wz+b\right)$$

where W is the weight matrix, and b is a bias vector. $score_{l}$ is an associated probability distribution of C class (C = 2). $score_{l}$ is the probability that the lncRNA $l_{1}$ is associated with the disease $d_{2}$ and $score_{l}^{0}$ is the probability that $l_{1}$ and $d_{2}$ have no association relationship. 

Similarly, the embedding matrix $Y\in R^{2\times \left(N_{l}+N_{m}+N_{d}\right)}$ is feed to the convolutional module on the right side of the prediction model for learning the network representation $Z_{right}$ of $l_{1}$ and $d_{2}$. $score_{r}$ of $l_{1}$ and $d_{2}$ are obtained when $Z_{right}$ is feed to the full connection layer and the softmax layer.

### 3.5. Convalutional Autoencoder Module on the Right

The matrices about lncRNA-disease association, lncRNA-miRNA interaction, and miRNA-disease association are very sparse, resulting in many 0 elements are contained in the embedding matrix Y. An autoencoder based convolutional neural network is constructed to learn important and low-dimensional feature representations of lncRNA-disease pair on the right side of CNNDLP. The encoding and decoding strategies are given as follows,

#### 3.5.1. Encoding Strategy

The embedding layer matrix $Y\in R^{2\times \left(N_{l}+N_{d}+N_{m}\right)}$ is mapped into the low-dimensional feature space through encoding based on convolutional neural network. $Y_{encode,k}^{\left(n-1\right)}$ is inputted to the *n-*th convolution layer to obtain $Z_{encode,k}^{\left(n\right)}$. $Y_{encode,k}^{\left(n\right)}$ is formed after $Z_{encode,k}^{\left(n\right)}$ passes the *n-*th max-pooling layer. They are defined as follows,

(12) $$Z_{encode,k}^{\left(n\right)}\left(i,j\right)=g\left(W_{encode,k}^{\left(n\right)}*Y_{encode,k}^{\left(n-1\right)}\left(i,j\right)+b_{encode}^{\left(n\right)}\left(k\right)\right)$$

(13) $$Y_{encode,k}^{\left(n\right)}\left(i,j\right)=max\left(Z_{encode,k}^{\left(n\right)}\left(i:i+w_{e},j+h_{e}\right)\right)$$

$$i\in \left[1,\;4-w_{e}+1\right],\;j\in \left[1,\;2+N_{l}+N_{m}+N_{d}-h_{e}+1\right],\;k\in \left[1,\;n_{encode}\right],\;n\in \left[1,H_{e}\right]$$

where $H_{e}$ is the total number of encoding layers, and $Y_{encode}^{\left(0\right)}=Y$. *k* represents the *k-*th filter and $n_{encode}$ is the number of filters during encoding process. $Z_{encode,k}^{\left(n\right)}\left(i,j\right)$ and $Y_{encode,k}^{\left(n\right)}\left(i,j\right)$ are the elements at the *i-*th row and the *j-*th column of the *k-*th feature map, respectively. $W_{encode,k}^{\left(n\right)}$ is a weight matrix and $b_{encode}^{\left(n\right)}\left(k\right)$ is a bias vector.

#### 3.5.2. Decoding Strategy

The output of the $H_{e}$-th encoding layer $Y_{encode}^{\left(H_{e}\right)}$ is used as the input of the decoder. It is a matrix that is similar to Y by decoding. The decoding process includes both the transpose convolution layer and transpose pooling layer, and they are respectively defined as,

(14) $$Z_{decode,k}^{\left(n\right)}\left(i,j\right)=g\left(W_{decode,k}^{\left(n\right)}*Y_{decode,k,i,j}^{\left(n-1\right)}+b_{decode}^{\left(n\right)}\left(k\right)\right)$$

(15) $$Y_{decode,k}^{\left(n\right)}\left(i,j\right)=Maxunpool\left(W_{decode,k}^{\left(n\right)}*Z_{decode,k}^{\left(n\right)}\left(i,j\right)+b_{decode}^{\left(n\right)}\left(k\right)\right)$$

$$\;k\in \left[1,\;n_{decode}\right],\;n\in \left[1,H_{d}\right]$$

$Y_{decode,k}^{\left(n\right)}$ and $Z_{decode,k}^{\left(n\right)}$ are the outputs of the *n-*th transpose convolution layer and transpose max-pooling layer, respectively. $H_{d}\;\mathrm{is}$ the total number of decoding layers, and $n_{decode}$ is the number of filters for decoding. As $Y_{decode}^{\left(H_{d}\right)}$ should be consistent with Y, we defined the loss function as follows,

(16) $$loss_{auto}=\frac{\sum_{i\;=\;1}^{T}{\left(Y_{i}-{\left(Y_{decode}\right)}_{i}\right)}^{2}}{T}$$

where $Y_{decode}$ is the output of decoding and Y is the input of encoding; $Y_{i}$ is corresponding to the *i-*th training sample (lncRNA-disease pair), and *T* is the number of training samples. The $score_{r}$ of $l_{1}$ and $d_{2}$ is obtained after the $Y_{encode}$ is feed to the full connection layer and the softmax layer.

### 3.6. Combined Strategy

In our model, the cross-entropy is used as the loss function, for the left and right parts of the prediction model loss functions are defined as follows,

(17) $$loss_{1}=-\sum_{i\;=\;1}^{T}\left[y_{label}\log\left(score_{l}\right)+\left(1-y_{label}\right)log\left(1-score_{l}\right)\right]$$

(18) $$loss_{2}=-\sum_{i\;=\;1}^{T}\left[y_{label}\log\left(score_{r}\right)+\left(1-y_{label}\right)log\left(1-score_{r}\right)\right]$$

where $y_{label}$ denotes the actual association label between a lncRNA and a disease. $y_{label}$ is 1 when the lncRNA is indeed associated with the disease, otherwise $y_{label}$ is 0. T is the number of training samples.

The final score of our model is a weighted sum of $score_{l}$ and $score_{r}$ as follows,

(19) $$score=\lambda score_{l}+\left(1-\lambda \right)score_{r}$$

where the parameter λ∈(0,1) is used to adjust the importance of $score_{l}$ and $score_{r}$.

## 4. Conclusions

A novel method based on the convolutional neural network with adjacent edge attention and convolutional autoencoder, entitled CNNDLP, is developed for inferring potential candidate lncRNA-disease associations. Two embedding layers are constructed from the biological perspective for integrating heterogeneous data about lncRNAs, diseases, and miRNAs from multiple sources. We construct the attention mechanism at the adjacent edge level to discriminate the different contributions of edges and the latent representation of a lncRNA-disease pair is learned from the more informative edges by the left side of CNNDLP’s prediction model. On the basis of calculating the new type of lncRNA similarity and that of disease similarity, the right side of CNNDLP’s model captures the complex relationships among these similarities and the lncRNA-disease associations, as well as the topological structures of multiple heterogeneous networks. The novel prediction model based on the convolutional neural network learns the attention representation and the low-dimensional network one of the lncRNA-disease pair. The experimental results demonstrated that CNNDLP outperforms the other methods in terms of not only AUCs but AUPRs as well. In particular, CNNDLP is more beneficial for the biologists as the top part of its ranking list may retrieve more real lncRNA-disease associations. Case studies on three diseases further confirm that CNNDLP is able to discover the potential candidate disease-related lncRNAs. CNNDLP may serve as a powerful prioritization tool that screens prospective candidates for the subsequent discovery of actual lncRNA-disease associations through wet-lab experiments.

## Figures and Tables

**Table 1:** AUCs of CNNDLP and other methods on all the diseases and 10 well-characterized diseases.

Disease Name	CNNDLP	Ping’s Method	AUC LDAP	SIMCLDA	MFLDA	CNNLDA
Prostate cancer	**0.951**	0.826	0.710	0.874	0.553	0.897
Stomach cancer	0.947	0.930	0.928	0.864	0.467	**0.958**
Lung cancer	**0.976**	0.911	0.882	0.790	0.676	0.940
Breast cancer	**0.956**	0.872	0.830	0.742	0.517	0.836
Reproduce organ cancer	**0.943**	0.818	0.742	0.707	0.740	0.922
Ovarian cancer	**0.970**	0.913	0.857	0.786	0.558	0.942
Hematologic cancer	**0.989**	0.908	0.903	0.828	0.716	0.934
Kidney cancer	**0.984**	0.979	0.977	0.728	0.677	0.956
Liver cancer	**0.956**	0.910	0.898	0.799	0.634	0.918
Thoracic cancer	**0.921**	0.860	0.792	0.792	0.649	0.890
Average AUC of 405 diseases	**0.969**	0.870	0.745	0.745	0.626	0.952

The bold values indicate the higher AUCs.

**Table 2:** AUPRs of CNNDLP and other methods on all the diseases and 10 well-characterized diseases.

Disease Name	CNNDLP	Ping’s Method	AUPR LDAP	SIMCLDA	MFLDA	CNNLDA
Prostate cancer	**0.538**	0.333	0.297	0.176	0.092	0.390
Stomach cancer	**0.373**	0.364	0.094	0.138	0.008	0.286
Lung cancer	**0.666**	0.437	0.363	0.131	0.171	0.058
Breast cancer	0.485	0.403	0.396	0.047	0.031	**0.964**
Reproduce organ cancer	**0.498**	0.281	0.240	0.130	0.103	0.091
Ovarian cancer	**0.552**	0.483	0.427	0.027	0.023	0.526
Hematologic cancer	**0.667**	0.403	0.370	0.216	0.121	0.523
Kidney cancer	0.569	**0.663**	0.462	0.030	0.034	0.584
Liver cancer	0.630	0.498	0.511	0.140	0.110	**0.666**
Thoracic cancer	**0.399**	0.383	0.364	0.155	0.102	0.890
Average AUC of 405 diseases	**0.286**	0.152	0.127	0.059	0.039	0.251

The bold values indicate the higher AUPRs.

**Table 3:** Comparing of different methods based on AUCs with the paired Wilcoxon test.

	SIMCLDA	Ping’s Method	MFLDA	LDAP	CNNLDA
*p*-value of ROC curve	9.2454 × 10^−6^	0.00048	5.9940 × 10^−7^	0.00121	0.00773
*p*-value of PR curve	8.3473 × 10^−7^	0.04174	3.5037 × 10^−8^	0.00126	0.00024

**Table 4:** The top 15 stomach cancer-related candidate lncRNAs.

Rank	lncRNA Name	Description	Rank	lncRNA Name	Description
1	SPRY4-IT1	Lnc2Cancer, LncRNADisease	9	CDKN2B-AS1	LncRNADisease
2	TINCR	Lnc2Cancer, LncRNADisease	10	CCAT1	Lnc2Cancer, LncRNADisease
3	H19	Lnc2Cancer, LncRNADisease	11	HOTAIR	Lnc2Cancer, LncRNADisease
4	TUSC7	Lnc2Cancer, LncRNADisease	12	GACAT2	LncRNADisease
5	BANCR	Lnc2Cancer, LncRNADisease	13	UCA1	Lnc2Cancer, LncRNADisease
6	MEG3	Lnc2Cancer, LncRNADisease	14	PVT1	Lnc2Cancer, LncRNADisease
7	GAS5	Lnc2Cancer, LncRNADisease	15	MEG8	literature
8	GHET1	Lnc2Cancer, LncRNADisease			

**Table 5:** The top 15 breast cancer-related candidate lncRNAs.

Rank	lncRNA Name	Description	Rank	lncRNA Name	Description
1	SOX2-OT	Lnc2Cancer, LncRNADisease	9	CCAT1	Lnc2Cancer, LncRNADisease
2	HOTAIR	Lnc2Cancer, LncRNADisease	10	GAS5	Lnc2Cancer, LncRNADisease
3	LINC00472	Lnc2Cancer, LncRNADisease	11	MIR124-2HG	literature
4	BCYRN1	LncRNADisease	12	XIST	Lnc2Cancer, LncRNADisease
5	LINC-PINT	literature	13	LINC-ROR	Lnc2Cancer, LncRNADisease
6	MALAT1	Lnc2Cancer, LncRNADisease	14	PANDAR	Lnc2Cancer, LncRNADisease
7	CDKN2B-AS1	LncRNADisease	15	AFAP1-AS1	Lnc2Cancer
8	SPRY4-IT1	Lnc2Cancer, LncRNADisease			

**Table 6:** The top 15 prostate cancer-related candidate lncRNAs.

Rank	lncRNA Name	Description	Rank	lncRNA Name	Description
1	CDKN2B-AS1	LncRNADisease	9	HOTAIR	Lnc2Cancer, LncRNADisease
2	PCGEM1	Lnc2Cancer, LncRNADisease	10	LINC00963	Lnc2Cancer, LncRNADisease
3	PVT1	Lnc2Cancer, LncRNADisease	11	H19	Lnc2Cancer, LncRNADisease
4	GAS5	Lnc2Cancer, LncRNADisease	12	MEG3	Lnc2Cancer, LncRNADisease
5	HOTTIP	Lnc2Cancer, LncRNADisease	13	TUG1	Lnc2Cancer, LncRNADisease
6	NEAT1	Lnc2Cancer, LncRNADisease	14	PCA3	Lnc2Cancer, LncRNADisease
7	PCAT5	Lnc2Cancer	15	DANCR	Lnc2Cancer, LncRNADisease
8	PRINS	Lnc2Cancer, LncRNADisease			

## Supplementary Materials

Supplementary materials can be found at https://www.mdpi.com/1422-0067/20/17/4260/s1.
