# Supplementary material for: CNNDLP: A Method Based on Convolutional Autoencoder and Convolutional Neural Network with Adjacent Edge Attention for Predicting lncRNA–Disease Associations
Source: Int J Mol Sci. 2019 Aug 30;20(17):4260. doi: 10.3390/ijms20174260 (PMC6747450; doi:10.3390/ijms20174260)
Supplement: Supplementary file 1 [file ijms-20-04260-s001.zip › Table S1.docx]

**Supplementary Table S1.** Accuracy and F1 score of CNNDLP during the five-fold cross-validation.

| Number of positive samples for training | Number of negative samples for training | Number of positive samples for testing | Number of negative samples for training | Accuracy | F1 score |
| --- | --- | --- | --- | --- | --- |
| 2149 | 2149 | 538 | 94513 | 0.9930 | 0.946 |
| 2149 | 2149 | 538 | 94513 | 0.9948 | 0.983 |
| 2150 | 2150 | 537 | 94513 | 0.9846 | 0.986 |
| 2150 | 2150 | 537 | 94513 | 0.9873 | 0.990 |
| 2150 | 2150 | 537 | 94513 | 0.9861 | 0.988 |
