# Supplementary material for: CNNDLP: A Method Based on Convolutional Autoencoder and Convolutional Neural Network with Adjacent Edge Attention for Predicting lncRNA–Disease Associations
Source: Int J Mol Sci. 2019 Aug 30;20(17):4260. doi: 10.3390/ijms20174260 (PMC6747450; doi:10.3390/ijms20174260)
Supplement: Supplementary file 1 [file ijms-20-04260-s001.zip › Table S2.docx]

**Supplementary Table S2.** The AUCs and AUPRs of CNNDLP for different values of $\alpha$.

| $\alpha$ | $\beta$ | AUC | AUPR |
| --- | --- | --- | --- |
| 0.1 | 0.8 | 0.846 | 0.185 |
| 0.2 | 0.8 | 0.908 | 0.228 |
| 0.3 | 0.8 | 0.896 | 0.235 |
| 0.4 | 0.8 | 0.923 | 0.263 |
| 0.5 | 0.8 | 0.935 | 0.281 |
| 0.6 | 0.8 | 0.948 | 0.274 |
| 0.7 | 0.8 | 0.950 | 0.276 |
| 0.8 | 0.8 | 0.956 | 0.280 |
| **0.9** | 0.8 | **0.969** | **0.286** |
