# Supplementary material for: CNNDLP: A Method Based on Convolutional Autoencoder and Convolutional Neural Network with Adjacent Edge Attention for Predicting lncRNA–Disease Associations
Source: Int J Mol Sci. 2019 Aug 30;20(17):4260. doi: 10.3390/ijms20174260 (PMC6747450; doi:10.3390/ijms20174260)
Supplement: Supplementary file 1 [file ijms-20-04260-s001.zip › Table S3.docx]

**Supplementary Table S3.** The AUCs and AUPRs of CNNDLP for different values of $\beta$.

| $\alpha$ | $\beta$ | AUC | AUPR |
| --- | --- | --- | --- |
| 0.9 | 0.1 | 0.882 | 0.203 |
| 0.9 | 0.2 | 0.902 | 0.232 |
| 0.9 | 0.3 | 0.903 | 0.235 |
| 0.9 | 0.4 | 0.918 | 0.233 |
| 0.9 | 0.5 | 0.943 | 0.246 |
| 0.9 | 0.6 | 0.950 | 0.272 |
| 0.9 | 0.7 | 0.958 | 0.279 |
| **0.9** | **0.8** | **0.969** | **0.286** |
| 0.9 | 0.9 | 0.966 | 0.274 |
