# Supplementary material for: CNNDLP: A Method Based on Convolutional Autoencoder and Convolutional Neural Network with Adjacent Edge Attention for Predicting lncRNA–Disease Associations
Source: Int J Mol Sci. 2019 Aug 30;20(17):4260. doi: 10.3390/ijms20174260 (PMC6747450; doi:10.3390/ijms20174260)
Supplement: Supplementary file 1 [file ijms-20-04260-s001.zip › Table S4.docx]

**Supplementary Table S4.** The AUCs and AUPRs of CNNDLP for different values of $\lambda$.

| $\lambda$ | AUC | AUPR | $\lambda$ | AUC | AUPR |
| --- | --- | --- | --- | --- | --- |
| 0.1 | 0.945 | 0.243 | 0.6 | 0.961 | 0.258 |
| 0.2 | 0.951 | 0.250 | 0.7 | 0.960 | 0.242 |
| **0.3** | **0.969** | **0.286** | 0.8 | 0.961 | 0.256 |
| 0.4 | 0.958 | 0.235 | 0.9 | 0.961 | 0.237 |
| 0.5 | 0.960 | 0.231 |  |  |  |
